# Differential Effects of Vpr on Single-cycle and Spreading HIV-1 Infections in CD4^+^ T-cells and Dendritic Cells

**DOI:** 10.1371/journal.pone.0035385

**Published:** 2012-05-03

**Authors:** Suresh de Silva, Vicente Planelles, Li Wu

**Affiliations:** 1 Center for Retrovirus Research, Department of Veterinary Biosciences, The Ohio State University, Columbus, Ohio, United States of America; 2 Division of Microbiology and Immunology, Department of Pathology, University of Utah, Salt Lake City, Utah, United States of America; Temple University, United States of America

## Abstract

The Vpr protein of human immunodeficiency virus type 1 (HIV-1) contributes to viral replication in non-dividing cells, specifically those of the myeloid lineage. However, the effects of Vpr in enhancing HIV-1 infection in dendritic cells have not been extensively investigated. Here, we evaluated the role of Vpr during infection of highly permissive peripheral blood mononuclear cells (PBMCs) and CD4^+^ T-cells and compared it to that of monocyte-derived dendritic cells (MDDCs), which are less susceptible to HIV-1 infection. Infections of dividing PBMCs and non-dividing MDDCs were carried out with single-cycle and replication-competent HIV-1 encoding intact Vpr or Vpr-defective mutants. In contrast to previous findings, we observed that single-cycle HIV-1 infection of both PBMCs and MDDCs was significantly enhanced in the presence of Vpr when the viral stocks were carefully characterized and titrated. HIV-1 DNA quantification revealed that Vpr only enhanced the reverse transcription and nuclear import processes in single-cycle HIV-1 infected MDDCs, but not in CD4^+^ T-cells. However, a significant enhancement in HIV-1 *gag* mRNA expression was observed in both CD4^+^ T-cells and MDDCs in the presence of Vpr. Furthermore, Vpr complementation into HIV-1 virions did not affect single-cycle viral infection of MDDCs, suggesting that newly synthesized Vpr plays a significant role to facilitate single-cycle HIV-1 infection. Over the course of a spreading infection, Vpr significantly enhanced replication-competent HIV-1 infection in MDDCs, while it modestly promoted viral infection in activated PBMCs. Quantification of viral DNA in replication-competent HIV-1 infected PBMCs and MDDCs revealed similar levels of reverse transcription products, but increased nuclear import in the presence of Vpr independent of the cell types. Taken together, our results suggest that Vpr has differential effects on single-cycle and spreading HIV-1 infections, which are dependent on the permissiveness of the target cell.

## Introduction

Among the four accessory proteins of HIV-1, the viral protein R (Vpr) has been widely investigated due to its efficient incorporation in the virion particle, its ability to alter the cell cycle, and its cytopathic nature (reviewed in [1], [2], [3]). Vpr is a small, 96-amino acid protein that is expressed in the infected cell from the provirus as a late viral gene product from a singly spliced mRNA [4], and is efficiently incorporated into the viral particle through its interaction with the C-terminal p6 region of the Gag precursor [5]. Due to its ability to interact with numerous cellular proteins [6], [7], several functions have been ascribed to Vpr. These include the induction of cell cycle arrest in the G2 phase [8], long-terminal-repeat (LTR)-transactivation [9], [10], [11], [12], induction of apoptosis [13], enhancement of the fidelity of reverse transcription [14], and impairment of host immune function for HIV-1 evasion [15], [16]. For instance, the Vpr-binding protein (VprBP), also called DDB1 (damaged DNA binding protein 1)- and Cullin-4 (Cul4)-associated factor 1 (DCAF1), is important for cell cycle regulation [7]. A current working model proposes that Vpr might be capable of targeting an unknown cell cycle regulatory factor for proteasomal degradation via the recruitment of the DDB1/DCAF1/Cul4A complex, which enables Vpr-mediated cell cycle arrest in the G2 phase of dividing cells [17], [18], [19], [20]. However, the role of DCAF1 in HIV-1 infection remains to be examined.

Another key function of Vpr is its requirement for HIV-1 infection in non-dividing cells such as macrophages *in vitro* [21], [22], [23], [24], [25]. It does so mainly by playing a chaperone-like role for importing the pre-integration complex containing the reverse transcribed viral DNA into the nucleus of the non-dividing cell, a function that is thought to be redundant in proliferating cells such as activated CD4^+^ T-cells where dissolution of the nuclear envelope occurs to facilitate integration of the viral genome [26]. Several reports suggest that other viral components such as capsid (CA) [27], [28], integrase (IN) [29], and the central DNA flap, which contains the polypurine tract-central termination sequence (cPPT-CTS) [30], [31], are required for nuclear import of viral DNA, especially in non-dividing cells. However, discrepancies exist with regard to the involvement of some of these viral factors in nuclear import [32], [33]. Recently, Rivière et al. performed a comprehensive analysis to identify which viral component among IN, Vpr, MA, and the cPPT-CTS was vital for the nuclear import of HIV-1 DNA in dividing and non-dividing cell types [30]. Using a vesicular stomatitis glycoprotein (VSV-G) pseudotyped, single-cycle HIV-1 vector devoid of all accessory genes, wherein the viral genes are under the transcriptional regulation of the cytomegalovirus promoter, they concluded that the cPPT-CTS of the DNA flap was most critical for nuclear import of viral DNA in peripheral blood lymphocytes, monocyte-derived dendritic cells (MDDCs) and macrophages [30]. They also reported that the Vpr-deleted mutant HIV-1 vector was similar to its Vpr-expressing counterpart in transduction of the three primary cell types that were tested and did not influence the nuclear import process. However, it is important to note that using a promoter-modified HIV-1 vector cannot fully reflect LTR promoter-driven viral replication and gene expression in infected cells.

To better understand the effects of Vpr on HIV-1 infection under highly permissive and less permissive cell type conditions, we examined the role of Vpr in activated peripheral blood mononucleocytes (PBMCs), CD4^+^ T-cells, and MDDCs in the context of single-cycle and replication-competent HIV-1 infections. Our results indicate that Vpr significantly enhances single-cycle HIV-1 infection in PBMCs, CD4^+^ T-cells and MDDCs. In contrast, Vpr significantly enhances replication of spreading HIV-1 infection in MDDCs, but not in PBMCs. Our data suggest distinct differences in the role of Vpr in single-cycle and replication-competent HIV-1 infection that are dependent on the permissiveness and cell cycle status of the target cell.

## Results

### Characterization of Vpr^+^ and Vpr^−^ Single-cycle, VSV-G-pseudotyped HIV-1 Stocks

**Figure 1:**
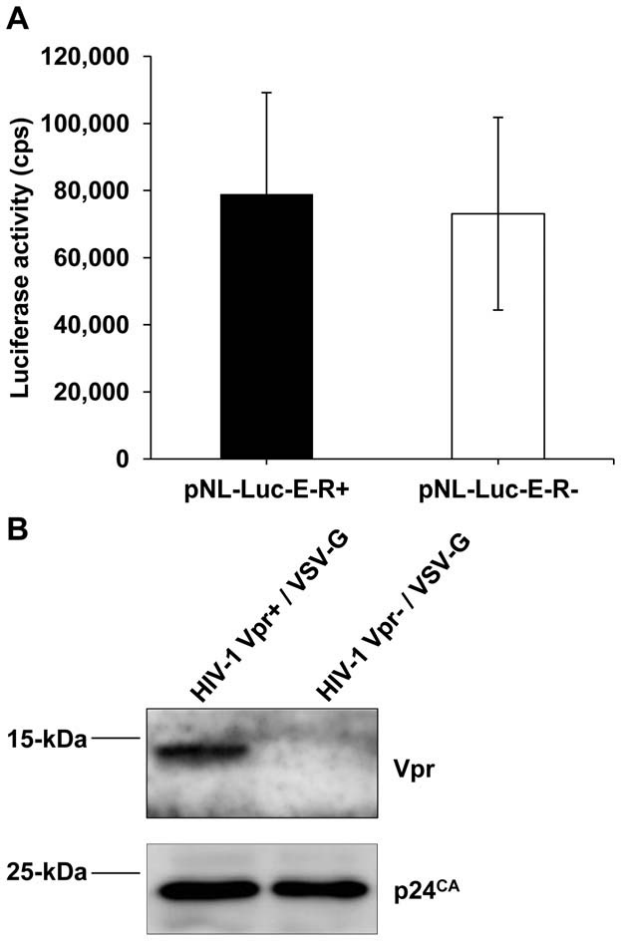
Characterization of Vpr^+^ and Vpr^−^ single-cycle, VSV-G-pseudotyped HIV-1 stocks produced from HEK293T cells. (A) Quantification of luciferase (Luc) expression from the pNL-Luc-E^−^R^+^ (HIV-1 Vpr^+^) and pNL-Luc-E^−^R^−^ (HIV-1 Vpr^−^) proviral DNA constructs in HEK293T cells. Luciferase activity was determined 48 h following plasmid transfection and normalized to protein content. Error bars represent the standard deviation of the mean of three independent experiments. (B) The VSV-G pseudotyped, HIV-1 Vpr^+^ and HIV-1 Vpr^−^ stocks generated from pNL-Luc-E^−^R^+^ and pNL-Luc-E^−^R^−^ were analyzed by immunoblotting for the incorporation of Vpr into virion particles.

To evaluate HIV-1 production and infectivity in the presence or absence of Vpr, we conducted a careful characterization and titration of the Vpr^+^ and Vpr^−^ virus stocks. The luciferase reporter HIV-1 proviral vectors NL-Luc-E^−^R^+^ (Vpr^+^) and the NL-Luc-E^−^R^−^ (Vpr^−^) were used for single-cycle virus production, which contain an intact Vpr open-reading-frame (ORF) or a frame-shift mutant of Vpr, respectively [21]. Prior to virus production, we confirmed that the frame-shift mutation in the Vpr ORF did not aberrantly affect the expression of the luciferase reporter gene by transfecting HEK293T cells with the pNL-Luc-E^−^R^+^ and pNL-Luc-E^−^R^−^ proviral plasmids and measuring luciferase reporter expression. Our results indicated that LTR-driven luciferase reporter gene expression from the *vpr*-deleted proviral plasmid was similar to its *vpr*-expressing counterpart (Fig. 1A). The proviral plasmids were then used to generate single-cycle HIV-1 pseudotyped with the VSV-G envelope from HEK293T cells. Similar levels of p24 capsid protein were detected in the two viral stocks (Fig. 1B and Table 1), which indicated that the lack of Vpr did not significantly affect virus production. Immunoblotting of lysed, HIV-1 Vpr^+^ and Vpr^−^ viral particles confirmed that Vpr was only incorporated in the HIV-1 Vpr^+^ due to an intact ORF contained within the NL-Luc-E^−^R^+^ vector (Fig. 1B).

**Table 1:** Titration of infectivity of single-cycle and replication-competent HIV-1 stocks.

HIV-1 stocks	p24 concentration (ng/ml)	Infectious titer (IU/ml)	Relative infectivity (IU/ng of p24)
Vpr^+^/VSV-G a	254	8.17×10^6^	3.29×10^4^
Vpr^−^/VSV-G a	173	6.34×10^6^	3.66×10^4^
Vpr^−^/VSV-G (Vpr complemented) a	114	8.66×10^6^	7.59×10^4^
Vpr^+^/Ampho b	305	7.69×10^5^	2.52×10^3^
Vpr^−^/Ampho b	309	6.77×10^5^	2.19×10^3^
NLAD8 WT c	720	2.26×10^7^	3.14×10^4^
NLAD8 ΔVpr c	680	1.91×10^7^	2.81×10^4^

a Single-cycle, VSV-G-pseudotyped luciferase reporter HIV-1 Vpr^+^, HIV-1 Vpr^−^, and Vpr complemented HIV-1 Vpr^−^ stocks. The data represent average results of duplicated samples from two independent experiments.

b Single-cycle, MLV amphotrophic (Ampho) envelope-pseudotyped luciferase reporter HIV-1 Vpr^+^ and HIV-1 Vpr^−^ virus stocks.

c Replication-competent HIV-1_NLAD8(WT)_ and HIV-1_NLAD8(ΔVpr)_ stocks. All viral stocks were prepared from HEK293T cells and analyzed for p24 concentration by ELISA. The infectivity of each virus stock was evaluated on HIV-1 indicator GHOST/R5 cells by a limiting dilution assay [35]. The relative infectivity of each virus stock is presented as the number of infectious units (IU) per 1 ng of p24.

To assess the infectivity of the single-cycle HIV-1 stocks, a limiting dilution infectivity assay was conducted on GHOST/R5 indicator cells and an infectious titer and relative infectivity was calculated for each virus stock. GHOST/R5 cells are human osteosarcoma cells that express CD4 and CCR5 and contain a GFP gene under the control of the HIV-2 LTR promoter, which is expressed during HIV-1 infection via Tat transactivation acting as an indicator of infection [34], [35], [36]. Our results confirmed that HIV-1 Vpr^+^ and HIV-1 Vpr^−^ were equally infectious in the GHOST/R5 indicator cell line (Table 1). Similar luciferase activities were obtained when HEK293T cells were infected with these single-cycle viruses (data not shown). Thus, Vpr expression does not significantly affect single-cycle HIV-1 production and virion infectivity.

### Vpr Enhances Single-cycle HIV-1 Infection of Activated PBMCs, Primary CD4^+^ T Cells and MDDCs

**Figure 2:**
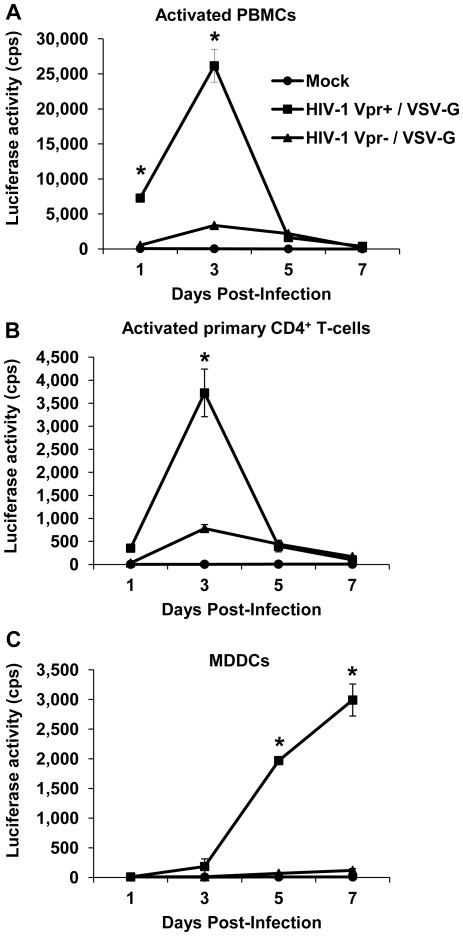
Vpr enhances single-cycle HIV-1 infection of activated PBMCs, primary CD4^+^ T cells, and MDDCs. (A) PHA-activated peripheral blood mononucleocytes (PBMCs), (B) PHA-activated CD4^+^ T cells, and (C) Monocyte-derived dendritic cells (MDDCs) were infected at an MOI of 1.0 with single-cycle HIV-1 Vpr^+^/VSV-G and HIV-1 Vpr^−^/VSV-G to test the role of Vpr in HIV-1 infection. Luciferase expression from the integrated provirus in the infected cells was assessed at the indicated time and normalized to protein content (10 µg/sample). The data shown represents one of three independent experiments carried out for each cell type from three different donors. Error bars represent the standard deviation of the mean of triplicate samples. Statistically significant differences are indicated by the asterisks (*P*<0.05).

To examine the role of Vpr in HIV-1 infection, we compared the infection of the two viruses on PHA-activated PBMCs, primary CD4^+^ T-cells, and MDDCs. Cells were separately infected with Vpr^+^ and Vpr^−^ single-cycle HIV-1 at an MOI of 1 and the level of infection was monitored over a 7-day period by measuring luciferase reporter expression. The infection of HIV-1 Vpr^+^ was robust in the highly permissive, activated PBMCs and CD4^+^ T-cells and the peak of luciferase expression was reached at 3 days post-infection (dpi) and declined sharply at 5 dpi (Fig. 2A and 2B). Such a sharp decline in luciferase expression in Vpr^+^ single cycle infected CD4^+^ primary T-cells has been previously reported and is attributed to Vpr induced inhibition of cell growth and/or cell death [37]. In contrast, HIV-1 Vpr^−^ failed to establish a robust infection in both primary cell types as evident by the amount of luciferase expressed from the infected cells at 3 and 5 dpi. At 3 dpi, the level of HIV-1 Vpr^+^ infection was 5-7-fold higher (*P*<0.05) than that of HIV-1 Vpr^−^ in PBMCs and CD4^+^ T-cells, respectively (Fig. 2A and 2B). In the less permissive, non-dividing MDDCs, infection of HIV-1 Vpr^+^ was 28-fold higher (*P*<0.05) than that of HIV-1 Vpr^−^ at 7 dpi (Fig. 2C). Furthermore, luciferase expression from HIV-1 Vpr^+^ infected MDDCs steadily increased over the 7-day period (Fig. 2C). However, the overall infection of HIV-1 Vpr^+^ in PBMCs was approximately 8-fold higher (*P*<0.05) than that in MDDCs (Fig. 2A and 2C). These results suggested that Vpr is required for efficient single-round HIV-1 infection of both permissive and less-permissive target cell types of HIV-1.

### Vpr-mediated Enhancement of Single-cycle HIV-1 Infection is Independent of VSV-G and Ampho Envelopes used for Virus Pseudotyping

**Figure 3:**
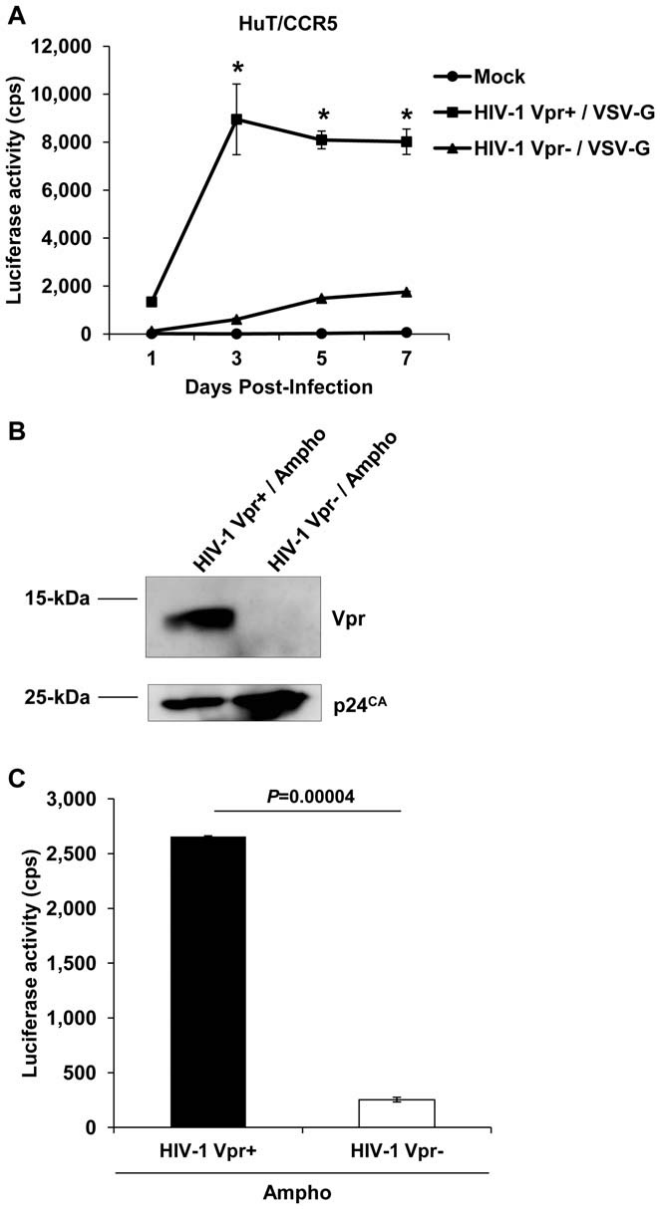
Vpr-mediated enhancement of single-cycle HIV-1 infection is independent of VSV-G and Ampho envelopes used for virus pseudotyping. (A) HuT/CCR5 cells were infected at an MOI of 1.0 with single-cycle HIV-1 Vpr^+^/VSV-G and HIV-1 Vpr^−^/VSV-G. Luciferase expression from the integrated provirus in the infected cells was assessed at the indicated time and normalized to protein content (10 µg/sample). The data shown represents one of three independent experiments, and error bars represent standard deviation of the mean of triplicate samples. (B) The MLV amphotrophic (Ampho) envelope pseudotyped, HIV-1 Vpr^+^ and HIV-1 Vpr^−^ stocks generated from pNL-Luc-E^−^R^+^ and pNL-Luc-E^−^R^−^ proviral constructs were analyzed by immunoblotting for the presence of Vpr. (C) HuT/CCR5 cells were infected at an MOI of 1.0 with HIV-1 Vpr^+^/Ampho and HIV-1 Vpr^−^/Ampho. Luciferase expression from the integrated provirus in the infected cells was assessed 3 days post infection and normalized to protein content. Error bars represent standard deviation of the mean of triplicate samples. Statistically significant differences are indicated by the asterisks (*P<*0.05) and the *P* value.

Next, we assessed whether a transformed T-cell line, such as CD4^+^ HuT/CCR5 cells [34], would still require the presence of Vpr to establish a robust single-cycle HIV-1 infection as observed with PBMCs and primary CD4^+^ T-cells. Accordingly, HuT/CCR5 cells were infected with VSV-G-pseudotyped Vpr^+^ and Vpr^−^ single-cycle HIV-1 at an MOI of 1, and infection was monitored by luciferase expression over a 7-day period. Similar to what was observed with CD4^+^ primary T-cells, the infection of HuT/CCR5 cells with Vpr^+^ HIV-1 was 15-fold higher (*P*<0.05) compared with Vpr^−^ HIV-1 infection (Fig. 3A). These results were unexpected as a previous study has shown that Vpr is not required for single-cycle HIV-1 infection in dividing cells [21].

To rule out the possibility that Vpr-mediated enhancement of HIV-1 infection was dependent on the type of envelope used for virus entry, NL-Luc-E^−^ single cycle virus stocks were generated using the same HIV-1 vectors but pseudotyped with the MLV amphotrophic envelope (Ampho), which has been used by previous studies of Vpr function [21], [37]. The HIV-1 Vpr^+^/Ampho and HIV-1 Vpr^−^/Ampho viral stocks were evaluated for the incorporation of Vpr in the virion by immunoblotting (Fig. 3B). HIV-1 p24 capsid concentration, infectious titer, and specific infectivity were examined (Table 1). Both virus stocks contained similar p24 levels and infected GHOST/R5 indicator cells in a similar manner. We then infected HuT/CCR5 cells with HIV-1 Vpr^+^/Ampho and HIV-1 Vpr^−^/Ampho stocks at an MOI of 1 and assessed luciferase expression at 3 dpi, since peak infection was reached at this time-point with the VSV-G pseudotyped virus infection (Fig. 3A). Our results indicated that the infection of HIV-1 Vpr^+^/Ampho was approximately 10-fold higher (*P = *0.00004) than the HIV-1 Vpr^−^/Ampho (Fig. 3C). These data suggest that single-cycle HIV-1 infection is enhanced significantly in the presence of Vpr independent of the type of heterologous virus envelope used for endocytosis-mediated virus entry.

### Quantification of the Levels of HIV-1 viral DNA Species Generated in Target Cells Following Single-cycle Infection

To identify at which step during the virus life cycle Vpr played a significant role to enhance single-cycle HIV-1 infection in both highly permissive and less permissive cell types, we performed real-time PCR analysis and quantified late reverse transcription (late RT) products, 2-LTR circles, and integrated copies of provirus following infection of the two cell types. Late RT products represent the full reverse transcribed viral DNA. Although 2-LTR circles produced from fully reverse-transcribed HIV-1 DNA are abortive products, they can be used as a surrogate marker for nuclear import of the viral DNA [38]. The amount of integrated proviral DNA in infected cells was quantified using *Alu-gag*-based real-time PCR [38]. Since we observed a distinct deficit in Vpr^−^ single-cycle HIV-1 infection in the HuT/CCR5 cell line (Fig. 3A), which was similar to what was observed in CD4^+^ primary T-cells (Fig. 2B), we performed quantitative PCR analysis in infected HuT/CCR5 cells and MDDCs.

**Figure 4:**
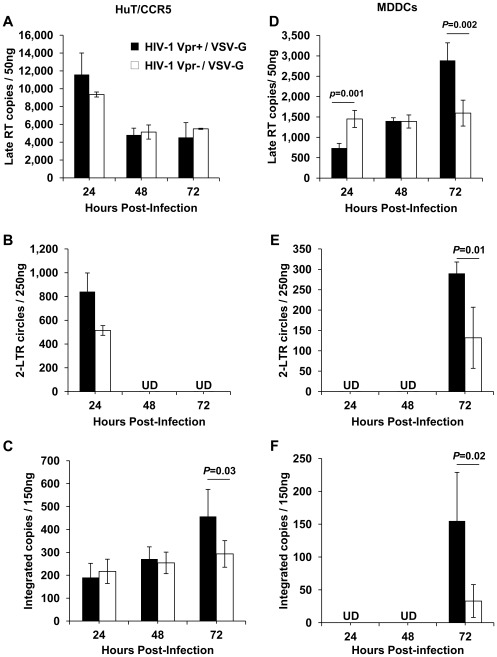
Comparison of the viral DNA profiles in single-cycle HIV-1 Vpr^+^/VSV-G and HIV-1 Vpr^−^/VSV-G infected cells. Cellular DNA was isolated from single cycle HIV-1 Vpr^+^/VSV-G and HIV-1 Vpr^−^/VSV-G infected HuT/CCR5 cells (A-C) and MDDCs (D-F) at 24, 48 and 72 h post-infection and subjected to real-time quantitative PCR analysis using Taqman-based primer/probe sets specific to quantify the levels of late-reverse transcription (Late-RT) products, 2-LTR circles, and integrated proviral copies. The amounts of genomic DNA used for the PCR are indicated in each panel. Real-time PCR amplification of the *glyceraldehyde-3-phosphate dehydrogenase* gene was performed for each sample to normalize for the amount of input DNA in each of the amplification reactions. Error bars represent standard error of the mean of duplicate samples. UD; undetectable under current experimental conditions. Statistically significant differences are indicated by *P* values. The MDDC data shown represents one of three independent experiments using cells from three different donors.

Our results indicate that the quantities of late RT products, which are generated upon completion of the reverse transcription process, were similar in HIV-1 Vpr^+^ and Vpr^−^ infected HuT/CCR5 cells and declined with similar kinetics over a 48-h time period beginning at 24 h post-infection (Fig. 4A). However, a modest, but not statistically significant increase (*P = *0.09) in the quantities of 2-LTR circles was observed at 24 h post-infection in HIV-1 Vpr^+^ infected HuT/CCR5 cells (Fig. 4B). These data suggest that nuclear import of HIV-1 DNA might be to some extent more efficient in these actively dividing cells in the presence of Vpr. However, the number of integrated proviral DNA copies was similar in HIV-1 Vpr^+^ and Vpr^−^ infected HuT/CCR5 cells at 24 and 48 h post-infection with a slight increase at 72 h in the presence of Vpr (Fig. 4C).

In the case of MDDCs, a steady increase in the quantities of late RT products was observed in HIV-1 Vpr^+^ infected cells over a 72-h time period following infection compared with the HIV-1 Vpr^−^ infected cells where a constant, relatively low level of late RT products were maintained (Fig. 4D). While 2-LTR circles and the number of integrated proviral DNA were only above the detection limit (10 copies) at 72 h post-infection, a result of slower infection kinetics and relatively lower level of infection in MDDCs compared with HuT/CCR5-cells, the level of 2-LTR and integrated viral species were higher in the presence of Vpr (Fig. 4E and 4F). These results suggest that Vpr plays a more important role in enhancing the reverse transcription and nuclear import processes of a single-round HIV-1 infection in MDDCs relative to HuT/CCR5 cells.

### Vpr Significantly Enhances HIV-1 *gag* mRNA Levels in HuT/CCR5 Cells and MDDCs

**Figure 5:**
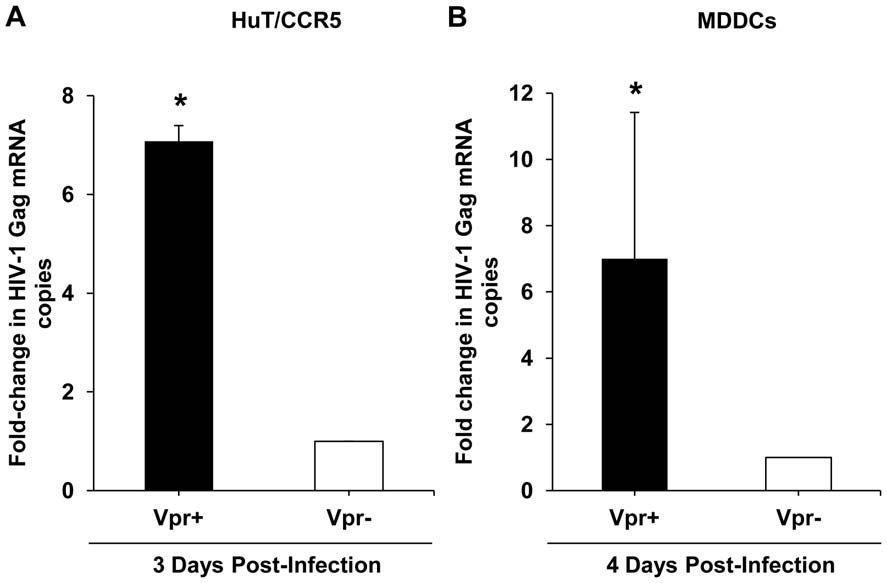
Vpr significantly enhances HIV-1 *gag* mRNA levels in HuT/CCR5 cells and MDDCs. Total cellular RNA was isolated from single cycle HIV-1 Vpr^+^/VSV-G and HIV-1 Vpr^−^/VSV-G infected HuT/CCR5 cells (A) and MDDCs (B) at 3 and 4 days post-infection, respectively, and subjected to RT-PCR to quantify the levels of HIV-1 *gag* mRNA copies in each cell type. The amplification of the *glyceraldehyde-3-phosphate dehydrogenase* gene was also performed for each sample to normalize for the amount of input cDNA in each of the amplification reactions. The data is represented as the fold change in the number of *gag* mRNA copies relative to the HIV-1 Vpr^−^/VSV-G infected sample in each cell type. Statistically significant differences are indicated by the asterisks (*P<*0.05). The MDDC data shown represents one of two independent experiments using cells from two different donors.

Vpr is a known transactivator of LTR-driven viral gene expression [9], [12], and thus, we questioned whether the increase in luciferase reporter gene expression from the integrated Vpr^+^ single cycle provirus compared with the Vpr^−^ provirus could also be due to Vpr-mediated increase in viral gene transcription. To this end total cellular RNA was extracted from HIV-1 Vpr^+^ and Vpr^−^ infected HuT/CCR5 cells and MDDCs, and subjected to real-time PCR to quantify the levels of HIV-1 *gag* mRNA produced from the infected cells. We quantified *gag* mRNA levels at 3 days post-infection in HuT/CCR5 cells and 4 days post-infection in MDDCs. These time points were chosen based on the reasoning that peak infection with single cycle HIV-1 occurred on or before these time points in the two cell types (Fig. 2B and 2C). We observed a significant increase (7-fold, *P* <0.05) in the number of *gag* mRNA copies in HIV-1 Vpr^+^ infected HuT/CCR5 cells and in MDDCs compared with the HIV-1 Vpr^−^ infected cells (Fig. 5A and 5B, respectively), which suggests that Vpr-mediated LTR transactivation is the likely cause for the enhancement in luciferase reporter expression from the infected in HuT/CCR5 cells and MDDCs.

### Knockdown of DCAF1 in HuT/CCR5 Cells does not Affect Single-cycle HIV-1 Infection

**Figure 6:**
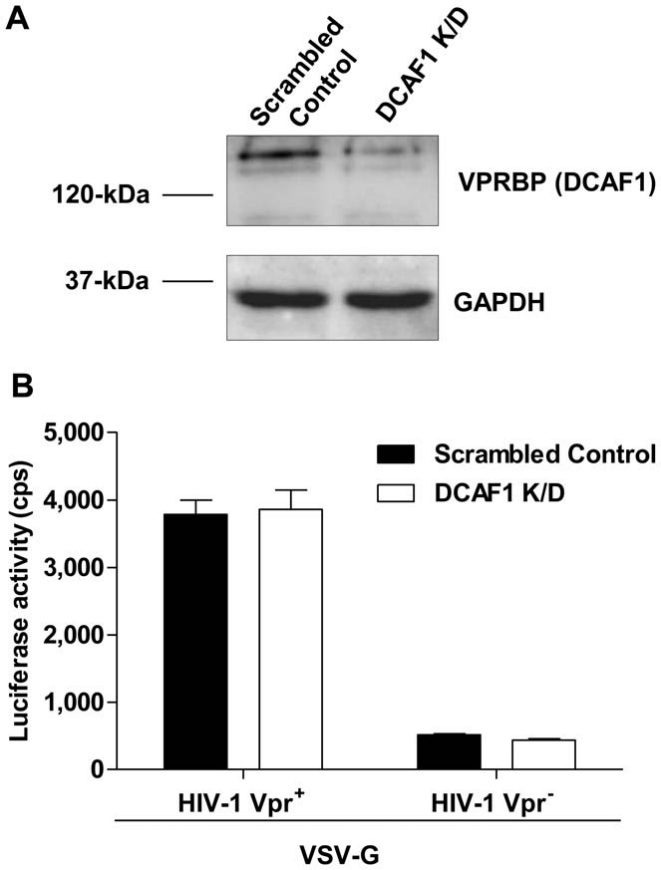
DCAF1 knockdown in HuT/CCR5 cells does not affect single-cycle HIV-1 infection. HuT/CCR5 cells were transduced with concentrated lentivirus expressing either shRNA targeting DCAF1 or a scrambled shRNA (control). Three days following transduction a fraction of cells were analyzed by immunoblotting to confirm DCAF1 knockdown (A), and cells each were infected at an MOI of 0.5 with either HIV-1 Vpr^+^/VSV-G or HIV-1 Vpr^−^/VSV-G to test whether DCAF1 was involved in the Vpr-mediated enhancement of HIV-1 infection (B). Luciferase expression in the infected cells was assessed at 3 days post infection.

To test whether the Vpr-mediated enhancement of single-cycle HIV-1 infection involved the DDB1/DCAF1/Cul4A complex and proteasomal degradation, DCAF1 was transiently knocked down in HuT/CCR5 cells using lentiviral vectors expressing shRNA-specific to DCAF1 and a scrambled shRNA vector was used as a control (Fig. 6A). The partial reduction in DCAF1 levels did not affect the infection of the HIV-1 Vpr^+^ and HIV-1 Vpr^+^ (Fig. 6A and 6B), suggesting that the Vpr-mediated enhancement of infection in HuT/CCR5 cells did not involve the recruitment of the DCAF1/DDB1/Cul4A complex and proteasomal degradation. Our result is consistent with a recent report by Pertel and colleagues, wherein they demonstrated that Vpr^+^ single-cycle HIV-1 infection of MDDCs was independent of DCAF1 [39].

### Vpr Complementation does not Affect Vpr-defective HIV-1 infection of MDDCs

**Figure 7:**
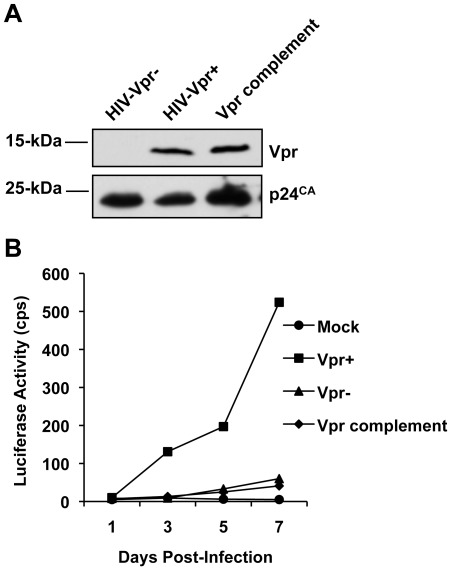
Vpr complementation does not affect Vpr-defective single-cycle HIV-1 infection of MDDCs. (A) Vpr incorporation in VSV-G-pseudotyped, single-cycle HIV-Vpr+ and Vpr-complemented HIV-Vpr-. Virion pellets were analyzed by immunoblotting with anti-Vpr and anti-p24, respectively. (B) Vpr complementation does not affect single-cycle HIV-Vpr- infection of MDDCs. Infected cells were lysed at indicated times post infection for the detection of HIV-1 infection by measuring luciferase activity and normalized to protein content (20 µg/sample). cps, counts per second. The data shown represents one of three independent experiments carried out with three individual donors.

Since high levels of Vpr can be incorporated into HIV-1 virions [5], we questioned whether the enhancement of HIV-1 infection of MDDCs resulted from incorporated Vpr into virions or newly synthesized Vpr in infected cells. To address this question, single-cycle HIV-1 with Vpr complementation was used in infection assays. High levels of Vpr were efficiently complemented into Vpr-negative single-cycle HIV-VSV-G as confirmed by immunoblotting (Fig. 7A). However, Vpr complementation did not affect single-cycle HIV-1 infection of MDDCs (Fig. 7B), suggesting that newly synthesized Vpr protein in infected MDDCs is required for efficient HIV-1 infection.

### Vpr Significantly Enhances Replication-competent HIV-1_NLAD8_ Infection in MDDCs

**Figure 8:**
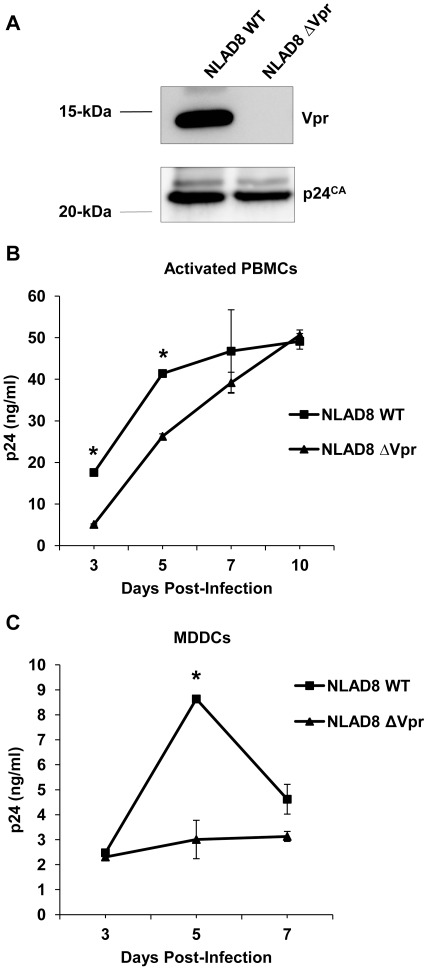
Vpr significantly enhances replication-competent HIV-1_NLAD8_ infection in MDDCs. (A) The HIV-1_NLAD8_ and HIV-1_NLAD8ΔVpr_ virus stocks produced from HEK293T cells were analyzed by immunoblotting for the presence of Vpr. (B) PHA-activated PBMCs and (C) MDDCs were infected with 5 ng and 20 ng of p24, respectively, from HIV-1_NLAD8_ and HIV-1_NLAD8ΔVpr_ virus and levels of p24 capsid released into the media during virus replication were assayed over a period of 10 days and 7 days post infection for PBMCs and MDDCs, respectively. The data shown represents one of three independent experiments carried out with three individual donors, and error bars represent standard deviation of triplicate infections. Statistically significant differences are indicated by the asterisks (*P<*0.05).

We next assessed whether Vpr of a replication-competent HIV-1 can enhance viral infection of PBMCs and MDDCs during multiple rounds of infection. Given that MDDCs are more susceptible to R5-tropic HIV-1 than to X4-tropic HIV-1 [40], [41], the R5-tropic strain HIV-1_NLAD8_ was used to compare the role of Vpr in HIV-1 infection. Individual virus stocks were evaluated for the incorporation of Vpr in the virion (Fig. 8A), p24 capsid concentration, infectious titer, and relative infectivity (Table 1). Comparable levels of p24 capsid protein were detected in the HIV-1_NLAD8_ WT and ΔVpr stocks and a limiting dilution infectivity assay on GHOST/R5 indicator cells confirmed that the ΔVpr virus was comparable in its infectivity to its WT counterpart (Table 1).

To assess the contribution of Vpr during multiple rounds of HIV-1 infection, PHA-stimulated PBMCs and MDDCs were infected with 5 ng and 20 ng of p24 from each virus type, respectively, and the level of infection was monitored over a 7- or 10-day period by quantifying the p24 production in the culture supernatant by ELISA. In activated PBMCs, the ΔVpr virus replicated at a slightly lower rate compared with the WT virus at 3 and 5 dpi (*P*<0.05), with both viruses displaying robust infections in activated PBMCs at 10 dpi (Fig. 8B). However, the disparity between the WT and ΔVpr virus was more far-reaching in MDDCs, where a 3-fold increase (*P*< 0.05) in infection was observed with the WT virus compared with the ΔVpr virus at 5 dpi (Fig. 8C), which is usually the peak of WT HIV-1 infection in MDDCs [42], [43]. These results suggest that during a spreading infection, the action of Vpr in enhancing HIV-1 replication in MDDCs compared with PBMCs is more readily observed.

### Comparison of the Viral DNA Profiles in HIV-1_NLAD8_ and HIV-1_NLAD8ΔVpr_ Infected Cells

**Figure 9:**
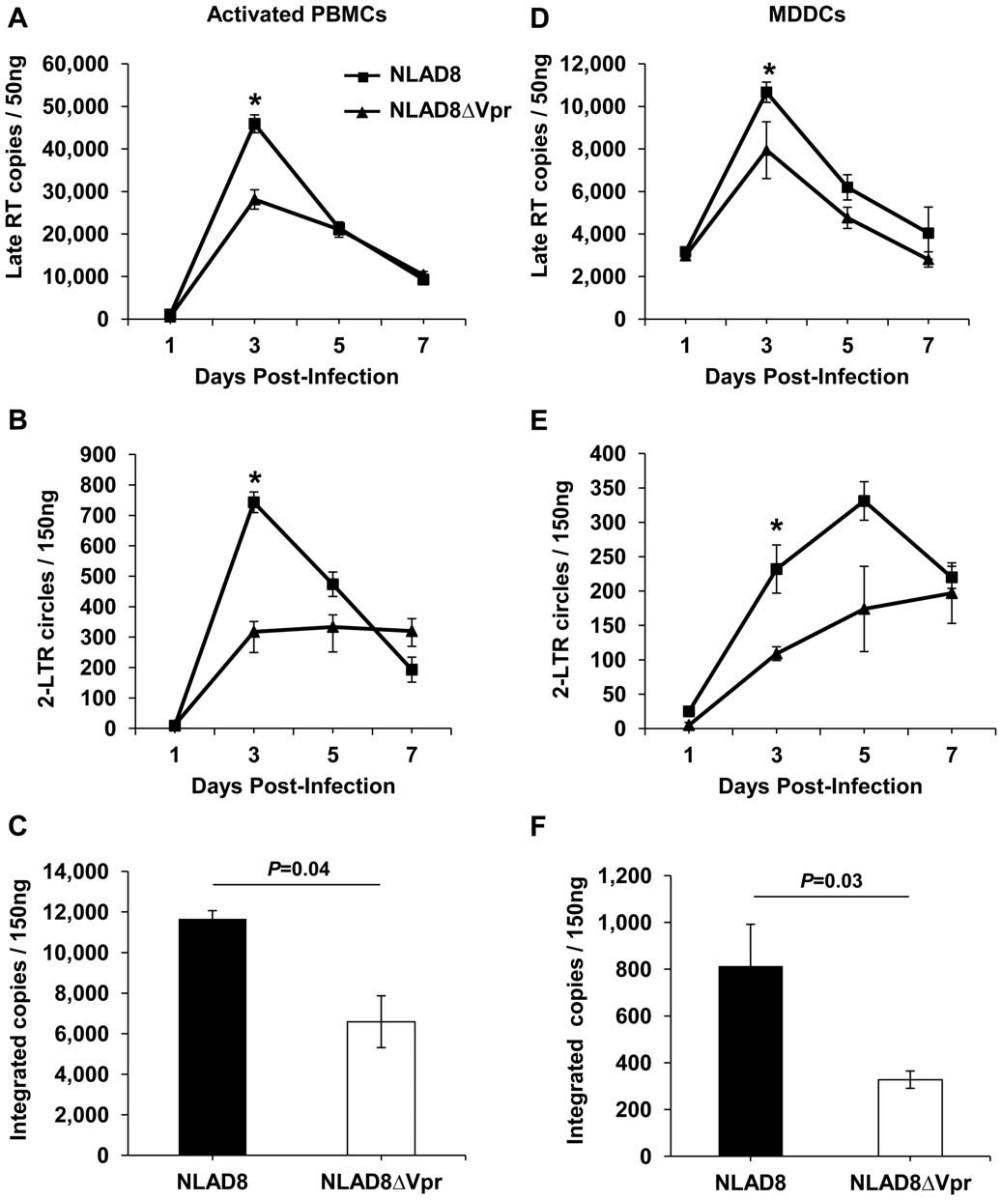
Comparison of the viral DNA profiles in HIV-1_NLAD8_ and HIV-1_NLAD8ΔVpr_ infected cells. Quantitative PCR analysis was performed to determine levels of late reverse transcription (Late-RT) products, 2-LTR circles, and integrated proviral copies over a period of 7 days following infection of activated PBMCs (A-C) and MDDCs (D-F) following infection with HIV-1_NLAD8_ and HIV-1_NLAD8ΔVpr_. Real-time PCR amplification of the *glyceraldehyde-3-phosphate dehydrogenase* gene was performed for each sample to normalize for the amount of input DNA in each of the amplification reactions. Error bars represent standard error of the mean of duplicate samples. Statistically significant differences are indicated by the asterisks (*P<*0.05) and *P* values. The data shown represents one of three independent experiments carried out for each cell type from three different donors.

To better understand the mechanisms by which Vpr enhances spreading infection, we performed quantitative PCR analysis on DNA isolated from either WT or ΔVpr HIV-1 infected PBMCs and MDDCs to determine the levels of late RT, 2-LTR circles and integrated proviral copies generated in the infected cells over multiple rounds of infection. Late RT products peaked at 3 dpi in both PBMCs and MDDCs with a modest increase in the presence of Vpr (*P*<0.05) (Fig. 9A and 9D). In both cell types, the levels of 2-LTR circles were higher in the presence of Vpr at 3 dpi (Fig. 9B and 9E). In addition, we measured integrated proviral copies at 3 dpi in PBMCs and observed a 1.8-fold increase (*P = *0.04) in the number of integrated copies in WT virus-infected cells compared with the ΔVpr virus infected cells (Fig. 9C). In a similar manner, the number of integrated proviral copies was higher in the presence of Vpr expression in MDDCs (2.5-fold, *P = *0.03) (Fig. 9F). As expected, the number of integrated proviral copies in MDDCs was 15-20-fold lower than in activated PBMCs (Fig. 9C and 9F). Collectively, our data indicate that Vpr increases nuclear import and integration of HIV-1 DNA in PBMCs and MDDCs, although ensuing virus production is significantly enhanced only in the less permissive MDDCs.

## Discussion

In the current study we compared the role of the HIV-1 accessory protein, Vpr, during single-cycle and replication-competent HIV-1 infection of PBMCs, CD4^+^ T-cells and MDDCs, cell types that are distinct with respect to their cell cycle status and susceptibility to HIV-1 infection. Single-cycle HIV-1 infection enables the study of viral and cellular factors affecting the HIV-1 virus life cycle to the point of viral DNA integration with the ensuing production of viral particles that lack envelope glycoprotein, thereby eliminating a second round of infection within the target cells. During single-cycle HIV-1 infection of PHA-activated primary PBMCs and CD4^+^ T-cells, we observed that Vpr significantly enhanced viral infection, which is in contrast to previously published results by Connor et al [21]. This initial study employed the same proviral constructs used in our study, but used the MLV amphotrophic envelope for virus pseudotyping. They showed that in the presence of polybrene, a cationic polymer that is used to enhance retroviral infection *in vitro*, the HIV-1 Vpr^−^ virus was able to infect activated PBMCs similar to the HIV-1 Vpr^+^ virus as determined by luciferase reporter expression. In contrast, we did not use polybrene and carefully titrated viral stocks in our infections. The discrepancy in results might be due to different experimental approaches including viral titration.

Given that PBMCs constitute a mixed population of mononuclear cells such as CD4^+^ and CD8^+^ T-cells, B-cells, monocytes, and dendritic cells etc., we isolated primary CD4^+^ T-cells from PBMCs and infected with single-cycle, Vpr^+^ and Vpr^−^ HIV-1. The results obtained from the primary CD4^+^ T-cells confirmed our findings in PBMCs and provided corroborative evidence that Vpr enhances single-cycle HIV-1 infection (Fig. 2B). Furthermore, pseudotyping of the single-cycle Vpr^−^ HIV-1 vector with an MLV amphotropic envelope displayed a similar defect in infection compared with the HIV-1 Vpr^+^/Ampho virus. Our results indicated that the lack of infectivity displayed by the single cycle, Vpr^−^ HIV-1 was due to a post-entry event independent of the type of heterologous virus envelope used for enodocytosis-mediated virus entry into the target cell.

Based on our quantitative PCR analysis of the viral DNA profiles in infected HuT/CCR5 cells, it appears that Vpr does not significantly affect the levels of late RT products, 2-LTR circles, and integration of viral DNA. Consistent with Vpr’s well-known ability to transactivate LTR-driven viral gene expression [9], [10], [25], we observed that *gag* mRNA levels was significantly increased in HIV-1 Vpr^+^ infected HuT/CCR5 cells compared to the HIV-1 Vpr^−^ infected cells, which appears to be the main contributing factor for the enhanced infection of single cycle HIV-1 expressing Vpr. Furthermore, our data indicated that DCAF1 is not required for Vpr-enhanced HIV-1 infection in HuT/CCR5 cells, which is consistent with a recent report demonstrating that single-cycle HIV-1 infection of MDDCs is independent of DCAF1 [39].

DCs share a common myeloid lineage as macrophages, which are also a target cell type of HIV-1 in initial viral infection, and have been implicated as possible viral reservoirs harboring latent HIV-1 virus [44]. Similar to DCs, macrophages are non-dividing cells, but are more permissive to HIV-1 provided Vpr is expressed from the HIV-1 genome [21], [22], [25]. Macrophages were the initial myeloid cell type to be used in studies, which indicated that Vpr was required for efficient nuclear import of HIV-1 DNA in non-dividing cell types [23], [25]. Thus, it is not surprising that DCs share the same requirement of Vpr for efficient HIV-1 infection. Interestingly, we found that in HIV-1 Vpr^+^ infected MDDCs, the quantities of late RT products steadily increased over the assessed time compared to HIV-1 Vpr^−^ infected cells (Fig. 4D). Given the slow infection kinetics in DCs, it is conceivable that the reverse transcription process occurs more efficiently in the presence of Vpr. Furthermore, based on the low level of infection that was achieved in MDDCs the number of 2-LTR circles and integrated proviral copies only exceeded the detection limit at 72 h post-infection with results indicating an increase in both viral DNA products in HIV-1 Vpr^+^ infected cells. Similar results have been reported previously in macrophages, which lead to the conclusion that Vpr enhanced nuclear import of viral DNA in non-dividing cells [23], [25]. However, we also observed an enhancement in *gag* mRNA levels in HIV-1 Vpr^+^ infected MDDCs compared with the HIV-1 Vpr^−^ infected cells, thus indicating that Vpr is capable of facilitating LTR-driven viral gene expression to enhance single cycle HIV-1 infection in DCs. Moreover, Vpr complementation into HIV-1 virions did not affect single-cycle HIV-1 infection of MDDCs, suggesting that Vpr-mediated enhancement of HIV-1 infection in MDDCs is exerted by the Vpr protein synthesized upon establishing infection, but not due to the Vpr protein associated with viral particles. Our results are in agreement with a previous study of Vpr in promoting HIV-1 infection of primary monocytes and macrophages [21].

Further confirmation for the role of Vpr in HIV-1 infection in DCs was obtained using replication-competent HIV-1 NLAD8(WT) and NLAD8(ΔVpr). However, Vpr did not seem to significantly enhance virus replication in activated PBMCs, which are more susceptible to spreading infection compared with MDDCs. This is consistent with an earlier study by Rey et al., wherein they reported an impairment of nuclear import of viral DNA in stimulated PBMCs in the absence of Vpr, which resulted in a subtle effect in virus production [45].

Cells of the myeloid lineage are inherently refractory to HIV-1 infection, which has been attributed to the presence of cellular restriction factors (reviewed in [46], [47]). The recent discovery of SAMHD1 as a cellular restriction factor of HIV-1 in myeloid cells provides evidence for such a notion [48], [49]. SAMHD1-mediated restriction of HIV-1 can be counteracted by the SIVmac/HIV-2 Vpx protein, which is absent in HIV-1. SIV/HIV-2 Vpx, but not HIV-1 Vpr, efficiently enhances SIV/HIV-1-derived lentiviral vector transduction of human monocytes, macrophages, or MDDCs (reviewed in [46]). HIV-1 and SIVmac Vpr cannot bind and degrade SAMHD1 [49], suggesting that Vpr is not able to counteract SAMHD1-mediated HIV-1 restriction in myeloid cells. Similar to several CD4^+^ T-cell lines [48], Hut/CCR5 cells do not express detectable SAMHD1 protein (data not shown). Despite the structural similarity between Vpx and Vpr from SIVmac, only Vpx, but not Vpr, can efficiently promote HIV-1 infection of human macrophages and the amino-terminal domain of Vpx is important for the enhancement of HIV-1 infection [50]. It remains to be determined whether the amino-terminal domain of HIV-1 Vpr is critical for its enhancement of viral infection. Moreover, APOBEC3A has been recently reported as a inhibitor of HIV-1 infection in myeloid cells [51]. Therefore, HIV-1 restriction in myeloid cell types may attribute to multiple host factors, which remains to be confirmed.

Our results indicate that Vpr enhances single-cycle and replication-competent HIV-1 infection in MDDCs. It remains unclear whether the enhancement in MDDCs is due to the counteraction of a cellular restriction factor by Vpr, although analysis of the different HIV-1 DNA in infected cells did not clearly indicate a restriction point. Furthermore, numerous cellular interacting partners of Vpr have been identified over the years, but none have been found to be restrictive of HIV-1 infection (reviewed in [7], [52]). It is possible that the Vpr-mediated enhancement of HIV-1 infection in MDDCs is not via the counteraction of a cellular restriction factor by Vpr, but merely by a synergistic effect on the different stages of the virus life cycle beginning with reverse transcription and ending with the regulation of viral genes. Further study of the mechanisms by which Vpr enhances HIV-1 infection will provide new insights into Vpr function in viral pathogenesis.

## Materials and Methods

### HIV-1 Stocks

Single-cycle, luciferase reporter HIV-1 stocks were generated by calcium phosphate-based transfection of HEK293T cells with the pNL-Luc-E^−^R^+^ proviral DNA vector, which contains an intact *vpr* gene or a frame-shift mutant of the *vpr* gene (pNL-Luc-E^−^R^−^), together with a construct expressing vesicular stomatitis virus glycoprotein (pVSV-G) [35] or murine leukemia virus (MLV) amphotrophic envelope glycoprotein (Ampho). Both proviral DNA constructs were kindly provided by Dr. Nathaniel Landau (New York University). Replication-competent HIV-1_NLAD8_ and HIV-1_NLAD8ΔVpr_ stocks were generated in HEK293T cells by calcium phosphate transfection of pNLAD8 and pNLAD8(ΔVpr), respectively [33] as described [43]. The p24 level in all virus stocks was determined using a p24 enzyme-linked immunosorbent assay (ELISA) kit (SAIC-Frederick) and the infectivity of each virus stock, represented as the infectious unit titer, was determined by limiting dilution on HIV-1 indicator GHOST/R5 cells as previously described [35].

### Immunoblotting

To confirm incorporation of Vpr in the virion particle in each virus stock, equivalent volumes of virus-containing media (1 ml) was ultracentrifuged at 35,000 rpm for 2 h at 4°C in a SW55 rotor. The virus pellet was lysed in 1×cell lysis buffer (Cell Signaling Technology) and subjected to immunoblotting using a polyclonal rabbit anti-Vpr antibody (the AIDS Research and Reference Reagent Program, NIH). Immunoblotting for p24 capsid protein was also conducted using a monoclonal mouse anti-p24 antibody as described [43] (clone #24-2, the AIDS Research and Reference Reagent Program, NIH).

### Cell Culture

PBMCs and monocytes were isolated from buffy coat from healthy blood donors by histopaque and percoll gradient centrifugation as previously described [40]. MDDCs were generated by treatment of monocytes with interleukin-4 (50 ng/ml) and granulocyte/macrophage-colony stimulating factor (50 ng/ml) for 5 days in culture. Primary CD4^+^ T cells were isolated from PBMCs using magnetic beads coated with CD4 antibodies (BD Biosciences). HEK293T, GHOST/R5, and Hut/CCR5 cell lines [34], [53] were kind gifts from Vineet KewalRamani (National Cancer Institute) and were maintained in specific media as previously described [34].

### HIV-1 Infection

For infections with single-cycle luciferase reporter HIV-1 viruses, MDDCs (2.5×10^5^) were infected at a multiplicity of infection (MOI) of 1 for 2 h at 37°C. Thereafter, the cells were washed twice in DPBS and cultured over a 7-day period. Activation of PBMCs and CD4^+^ T-cells were carried out with phytohemagglutinin (PHA; 5 µg/ml) and IL-2 (20 U/ml) for 24 h prior to infection with single cycle luciferase-reporter HIV-1 at an MOI of 1. At day 1, 3, 5 and 7 post-infection, cells were harvested, lysed in 1×reporter lysis buffer (Promega), and luciferase activity was detected using a commercially available kit (Promega). Infection of MDDCs with replication-competent HIV-1_NLAD8_ virus was conducted in a similar manner to the single cycle virus infections with 2.5×10^5^ PHA-activated PBMCs or MDDCs infected with 5 ng and 20 ng of p24, respectively. Gag p24 released into the culture supernatant during the infection period was assessed by ELISA as previously described [40].

### Quantitative PCR Analysis

Levels of late reverse transcription products, 2-LTR circles and integrated copies of provirus in infected MDDCs and activated PBMCs were quantified by Taqman-based real-time quantitative PCR analysis using primer and probe sets and protocols previously described [38]. Specifically, 50 ng of genomic DNA from HIV-1 infected cells was used as input for the detection of late reverse transcription products, and 150-250 ng for the detection of 2-LTR circles and integrated proviral DNA. All virus stocks were treated with DNaseI (40 U/ml; Ambion) prior to infections to avoid plasmid DNA contamination. DNA from infected cells at various time points was isolated using a DNeasy Blood and Tissue kit (QIAgen).

### RT-PCR Detection of *gag* mRNA

HuT/CCR5 cells and MDDCs (2.5×10^5^ cells) were infected with either HIV-1 Vpr^+^/VSV-G or HIV-1 Vpr^−^/VSV-G (MOI of 1) and harvested on day 3 (for HuT/CCR5 cells) and day 4 (for MDDCs) post-infection. Total cellular RNA was isolated using an RNeasy Mini kit (Invitrogen), and 250 ng of RNA was used as template for first strand cDNA synthesis using a Superscript III first-strand synthesis kit and oligo-dT primers (Invitrogen). Sybergreen-based real-time PCR analysis was performed using *gag*-specific primers [38] to quantify the levels of HIV-1 *gag* mRNA copies in each cell type. As standards for real-time PCR, serial dilutions (10^6^ to 10^2^ copies) of the pNLAD8 plasmid were used for the *gag* reaction. The amplification of *glyceraldehyde-3-phosphate dehydrogenase* cDNA was also performed for each sample to normalize for the amount of input cDNA in each of the amplification reactions.

### Lentivirus-mediated Knockdown of DCAF1 in Hut/CCR5 Cells

Lentivirus required for the shRNA-mediated knockdown of DCAF1 was generated by calcium phosphate-based co-transfection of HEK293T cells with a DCAF-1-specific shRNA-expressing lentiviral vector (pFG.12.3590) together with a packaging vector (pCVM) and a VSV-G expressing vector (p-VSV-G). Two days post-transfection, the media containing lentivirus were harvested, spun down to remove cellular debris and concentrated 5-fold using a 300,000 MW cut-off VIVASPIN 20 concentrator (Sartorius Stedim). Thereafter, the concentrated lentivirus was incubated with 2×10^6^ HuT/CCR5 cells together with 10 µg/ml polybrene for 2 h at 37°C. Following incubation, the cells were washed twice in 1X DPBS and re-plated in HuT/CCR5 cell media and cultured for 3 days and the knockdown of DCAF1 protein level was confirmed by immunoblotting using a rabbit polyclonal anti-VPRBP (DCAF-1) antibody (Proteintech).

### Vpr Complementation in Vpr-defective Single-cycle HIV-1

To generate single-cycle HIV-Luc/VSV-G complemented with Vpr, HEK293T cells were cotransfected with a Vpr-expressing construct (pcDNA-Vpr), pNL-Luc-E^–^R^–^, and pVSV-G to complement Vpr into Vpr-negative viruses as previously described [21]. The empty vector pcDNA was used as a negative control in the transfection.

### Statistical Analyses

Statistical analyses were performed using the Student’s *t*-test with the Excel program. Statistical significance was defined as *P*<0.05.

## References

[1] Le Rouzic E, Benichou S (2005). The Vpr protein from HIV-1: distinct roles along the viral life cycle.. Retrovirology.

[2] Andersen JL, Planelles V (2005). The role of Vpr in HIV-1 pathogenesis.. Curr HIV Res.

[3] Majumder B, Venkatachari NJ, Srinivasan A, Ayyavoo V (2009). HIV-1 mediated immune pathogenesis: spotlight on the role of viral protein R (Vpr).. Curr HIV Res.

[4] Schwartz S, Felber BK, Pavlakis GN (1991). Expression of human immunodeficiency virus type 1 vif and vpr mRNAs is Rev-dependent and regulated by splicing.. Virology.

[5] Paxton W, Connor RI, Landau NR (1993). Incorporation of Vpr into human immunodeficiency virus type 1 virions: requirement for the p6 region of gag and mutational analysis.. J Virol.

[6] Lama J, Planelles V (2007). Host factors influencing susceptibility to HIV infection and AIDS progression.. Retrovirology.

[7] Planelles V, Benichou S (2009). Vpr and its interactions with cellular proteins.. Current Topics in Microbiology and Immunology.

[8] Rogel ME, Wu LI, Emerman M (1995). The human immunodeficiency virus type 1 vpr gene prevents cell proliferation during chronic infection.. Journal of Virology.

[9] Gummuluru S, Emerman M (1999). Cell cycle- and Vpr-mediated regulation of human immunodeficiency virus type 1 expression in primary and transformed T-cell lines.. J Virol.

[10] Goh WC, Rogel ME, Kinsey CM, Michael SF, Fultz PN (1998). HIV-1 Vpr increases viral expression by manipulation of the cell cycle: a mechanism for selection of Vpr in vivo.. Nature Medicine.

[11] Agostini I, Navarro JM, Rey F, Bouhamdan M, Spire B (1996). The human immunodeficiency virus type 1 Vpr transactivator: cooperation with promoter-bound activator domains and binding to TFIIB.. J Mol Biol.

[12] Zhu Y, Gelbard HA, Roshal M, Pursell S, Jamieson BD (2001). Comparison of cell cycle arrest, transactivation, and apoptosis induced by the simian immunodeficiency virus SIVagm and human immunodeficiency virus type 1 vpr genes.. J Virol.

[13] Arokium H, Kamata M, Chen I (2009). Virion-associated Vpr of human immunodeficiency virus type 1 triggers activation of apoptotic events and enhances fas-induced apoptosis in human T cells.. J Virol.

[14] Mansky LM, Preveral S, Selig L, Benarous R, Benichou S (2000). The interaction of vpr with uracil DNA glycosylase modulates the human immunodeficiency virus type 1 In vivo mutation rate.. J Virol.

[15] Majumder B, Janket ML, Schafer EA, Schaubert K, Huang XL (2005). Human immunodeficiency virus type 1 Vpr impairs dendritic cell maturation and T-cell activation: implications for viral immune escape.. J Virol.

[16] Ayyavoo V, Mahboubi A, Mahalingam S, Ramalingam R, Kudchodkar S (1997). HIV-1 Vpr suppresses immune activation and apoptosis through regulation of nuclear factor kappa B. Nature Medicine.

[17] Dehart JL, Planelles V (2008). Human immunodeficiency virus type 1 Vpr links proteasomal degradation and checkpoint activation.. J Virol.

[18] DeHart JL, Zimmerman ES, Ardon O, Monteiro-Filho CM, Arganaraz ER, et al (2007). HIV-1 Vpr activates the G2 checkpoint through manipulation of the ubiquitin proteasome system.. Virol J.

[19] Belzile JP, Duisit G, Rougeau N, Mercier J, Finzi A (2007). HIV-1 Vpr-mediated G2 arrest involves the DDB1-CUL4AVPRBP E3 ubiquitin ligase.. PLoS Pathog.

[20] Hrecka K, Gierszewska M, Srivastava S, Kozaczkiewicz L, Swanson SK (2007). Lentiviral Vpr usurps Cul4-DDB1[VprBP] E3 ubiquitin ligase to modulate cell cycle.. Proc Natl Acad Sci U S A.

[21] Connor RI, Chen BK, Choe S, Landau NR (1995). Vpr is required for efficient replication of human immunodeficiency virus type-1 in mononuclear phagocytes.. Virology.

[22] Balliet JW, Kolson DL, Eiger G, Kim FM, McGann KA (1994). Distinct effects in primary macrophages and lymphocytes of the human immunodeficiency virus type 1 accessory genes vpr, vpu, and nef: mutational analysis of a primary HIV-1 isolate.. Virology.

[23] Heinzinger NK, Bukinsky MI, Haggerty SA, Ragland AM, Kewalramani V, et al (1994). The Vpr protein of human immunodeficiency virus type 1 influences nuclear localization of viral nucleic acids in nondividing host cells.. Proc Natl Acad Sci U S A.

[24] Vodicka MA, Koepp DM, Silver PA, Emerman M (1998). HIV-1 Vpr interacts with the nuclear transport pathway to promote macrophage infection.. Genes Dev.

[25] Subbramanian RA, Kessous-Elbaz A, Lodge R, Forget J, Yao XJ (1998). Human immunodeficiency virus type 1 Vpr is a positive regulator of viral transcription and infectivity in primary human macrophages.. J Exp Med.

[26] Dedera D, Hu W, Vander Heyden N, Ratner L (1989). Viral protein R of human immunodeficiency virus types 1 and 2 is dispensable for replication and cytopathogenicity in lymphoid cells.. J Virol.

[27] Yamashita M, Perez O, Hope TJ, Emerman M (2007). Evidence for direct involvement of the capsid protein in HIV infection of nondividing cells.. PLoS Pathogens.

[28] Yamashita M, Emerman M (2009). Cellular restriction targeting viral capsids perturbs human immunodeficiency virus type 1 infection of nondividing cells.. Journal of Virology.

[29] Gallay P, Hope T, Chin D, Trono D (1997). HIV-1 infection of nondividing cells through the recognition of integrase by the importin/karyopherin pathway.. Proc Natl Acad Sci U S A.

[30] Riviere L, Darlix JL, Cimarelli A (2010). Analysis of the viral elements required in the nuclear import of HIV-1 DNA.. J Virol.

[31] Ao Z, Yao X, Cohen EA (2004). Assessment of the role of the central DNA flap in human immunodeficiency virus type 1 replication by using a single-cycle replication system.. J Virol.

[32] Yamashita M, Emerman M (2005). The cell cycle independence of HIV infections is not determined by known karyophilic viral elements.. PLoS Pathogens.

[33] Freed EO, Englund G, Martin MA (1995). Role of the basic domain of human immunodeficiency virus type 1 matrix in macrophage infection.. J Virol.

[34] Wu L, Martin TD, Vazeux R, Unutmaz D, KewalRamani VN (2002). Functional evaluation of DC-SIGN monoclonal antibodies reveals DC-SIGN interactions with ICAM-3 do not promote human immunodeficiency virus type 1 transmission.. J Virol.

[35] Janas AM, Wu L (2009). HIV-1 interactions with cells: from viral binding to cell-cell transmission.. Curr Protoc Cell Biol Chapter 26: Unit 26.

[36] Cecilia D, KewalRamani VN, O'Leary J, Volsky B, Nyambi P (1998). Neutralization profiles of primary human immunodeficiency virus type 1 isolates in the context of coreceptor usage.. J Virol.

[37] Planelles V, Bachelerie F, Jowett JB, Haislip A, Xie Y (1995). Fate of the human immunodeficiency virus type 1 provirus in infected cells: a role for vpr.. J Virol.

[38] Dong C, Janas AM, Wang JH, Olson WJ, Wu L (2007). Characterization of human immunodeficiency virus type 1 replication in immature and mature dendritic cells reveals dissociable cis- and trans-infection.. J Virol.

[39] Pertel T, Reinhard C, Luban J (2011). Vpx rescues HIV-1 transduction of dendritic cells from the antiviral state established by type 1 interferon.. Retrovirology.

[40] Wang JH, Janas AM, Olson WJ, KewalRamani VN, Wu L (2007). CD4 coexpression regulates DC-SIGN-mediated transmission of human immunodeficiency virus type 1.. J Virol.

[41] Smed-Sorensen A, Lore K, Vasudevan J, Louder MK, Andersson J (2005). Differential susceptibility to human immunodeficiency virus type 1 infection of myeloid and plasmacytoid dendritic cells.. J Virol.

[42] Wang JH, Janas AM, Olson WJ, Wu L (2007). Functionally distinct transmission of human immunodeficiency virus type 1 mediated by immature and mature dendritic cells.. J Virol.

[43] Coleman CM, Spearman P, Wu L (2011). Tetherin does not significantly restrict dendritic cell-mediated HIV-1 transmission and its expression is upregulated by newly synthesized HIV-1 Nef.. Retrovirology.

[44] Coleman CM, Wu L (2009). HIV interactions with monocytes and dendritic cells: viral latency and reservoirs.. Retrovirology.

[45] Rey F, BouHamdan M, Navarro JM, Agostini I, Willetts K (1998). A role for human immunodeficiency virus type 1 Vpr during infection of peripheral blood mononuclear cells.. J Gen Virol 79 (Pt.

[46] Ayinde D, Maudet C, Transy C, Margottin-Goguet F (2010). Limelight on two HIV/SIV accessory proteins in macrophage infection: is Vpx overshadowing Vpr?. Retrovirology.

[47] St Gelais C, Wu L (2011). SAMHD1: a new insight into HIV-1 restriction in myeloid cells.. Retrovirology.

[48] Laguette N, Sobhian B, Casartelli N, Ringeard M, Chable-Bessia C (2011). SAMHD1 is the dendritic- and myeloid-cell-specific HIV-1 restriction factor counteracted by Vpx.. Nature.

[49] Hrecka K, Hao C, Gierszewska M, Swanson SK, Kesik-Brodacka M (2011). Vpx relieves inhibition of HIV-1 infection of macrophages mediated by the SAMHD1 protein.. Nature.

[50] Gramberg T, Sunseri N, Landau NR (2010). Evidence for an activation domain at the amino terminus of simian immunodeficiency virus Vpx.. Journal of Virology.

[51] Berger G, Durand S, Fargier G, Nguyen XN, Cordeil S (2011). APOBEC3A Is a Specific Inhibitor of the Early Phases of HIV-1 Infection in Myeloid Cells.. PLoS Pathog.

[52] Planelles V (2011). Restricted access to myeloid cells explained.. Viruses.

[53] Wu L, Martin TD, Carrington M, KewalRamani VN (2004). Raji B cells, misidentified as THP-1 cells, stimulate DC-SIGN-mediated HIV transmission.. Virology.

